# Gut Microbial Dysbiosis Is Associated with Altered Hepatic Functions and Serum Metabolites in Chronic Hepatitis B Patients

**DOI:** 10.3389/fmicb.2017.02222

**Published:** 2017-11-13

**Authors:** Jing Wang, Yang Wang, Xu Zhang, Jiaqi Liu, Qianpeng Zhang, Yu Zhao, Jinghua Peng, Qin Feng, Jianye Dai, Shujun Sun, Yufeng Zhao, Liping Zhao, Yongyu Zhang, Yiyang Hu, Menghui Zhang

**Affiliations:** ^1^State Key Laboratory of Microbial Metabolism, Joint International Research Laboratory of Metabolic and Developmental Sciences, and School of Life Sciences and Biotechnology, Shanghai Jiao Tong University, Shanghai, China; ^2^Center for Traditional Chinese Medicine and Systems Biology, Shanghai University of Traditional Chinese Medicine, Shanghai, China; ^3^Institute of Liver Disease, Shuguang Hospital, Shanghai University of Traditional Chinese Medicine, Shanghai, China; ^4^Department of Chemical Biology, College of Chemistry and Molecular Engineering, Peking University, Beijing, China; ^5^School of Pharmacy, Shanghai Jiao Tong University, Shanghai, China; ^6^Unimicro (Shanghai) Technologics Co., Ltd., Shanghai, China; ^7^School of Traditional Dai Medicine, West Yunnan University of Applied Sciences, Jinghong, Yunnan, China

**Keywords:** chronic hepatitis B, gut sysbiosis, 16S rRNA gene sequencing, serum metabolomics, aromatic amino acids

## Abstract

Chronic hepatitis B (CHB) is a global epidemic disease that results from hepatitis B virus (HBV) infection and may progress to severe liver failure, including liver fibrosis, cirrhosis and hepatocellular carcinoma. Previous evidence has indicated that the dysbiosis of gut microbiota occurs after liver virus infection and is associated with severe liver disease. The aim of this study is to elucidate the compositional and functional characteristics of the gut microbiota in early-stage CHB and to understand their influence on disease progression. We investigated the gut microbial composition of stool samples from 85 CHB patients with low Child-Pugh scores and 22 healthy controls using the Illumina MiSeq sequencing platform. Furthermore, the serum metabolome of 40 subjects was measured by gas chromatography mass spectrometry. Compared with the controls, significant alteration in the gut microbiota was observed in the CHB patients; 5 operational taxonomic units (OTUs) belonging to *Actinomyces, Clostridium sensu stricto*, unclassified Lachnospiraceae and *Megamonas* were increased, and 27 belonging to *Alistipes, Asaccharobacter, Bacteroides, Butyricimonas, Clostridium IV, Escherichia/Shigella, Parabacteroides, Ruminococcus*, unclassified Bacteria, unclassified Clostridiales, Unclassified Coriobacteriaceae, unclassified Enterobacteriaceae, unclassified Lachnospiraceae and unclassified Ruminococcaceae were decreased. The inferred metagenomic information of gut microbiota in CHB showed 21 enriched and 17 depleted KEGG level-2 pathways. Four OTUs, OTU38 (*Streptococcus*), OTU124 (*Veillonella*), OTU224 (*Streptococcus*), and OTU55 (*Haemophilus*), had high correlations with hosts' hepatic function indices and 10 serum metabolites, including phenylalanine and tyrosine, which are aromatic amino acids that play pathogenic roles in liver disease. In particular, these 4 OTUs were significantly higher in patients with higher Child-Pugh scores, who also showed diminished phenylalanine and tryptophan metabolisms in the inferred gut metagenomic functions. These compositional and functional changes in the gut microbiota in early-stage CHB patients suggest the potential contributions of gut microbiota to the progression of CHB, and thus provide new insight into gut microbiota-targeted interventions to improve the prognosis of this disease.

## Introduction

Chronic hepatitis B (CHB) is a globally prevalent disease estimated to affect 240 million individuals in 2005 (Ott et al., 2012). As a consequence of hepatitis B virus (HBV) infection, CHB is the primary contributor to liver fibrosis, cirrhosis and hepatocellular carcinoma (HCC), the latter two of which cause ~310,000 and ~340,000 deaths annually worldwide, respectively (Lozano et al., 2012). The systematic monitoring of CHB patients and delaying disease progression to severe liver failure is urgently needed (Terrault et al., 2015).

Interactions between gut microbiota and liver disease have received considerable attention. On the one hand, liver metabolites, such as bile acids, influence the microbial composition of the gut through the hepatic portal and bile secretion systems (Begley et al., 2005; Schnabl and Brenner, 2014). On the other hand, Gut et al. revealed that germ-free mice could be protected from virus-induced liver disease, suggesting the pathogenic role of gut microbes (Gut et al., 1984). Gut microbiota dysbiosis was found in HBV-infected patients with an increase in bacterial translocation and endotoxin load in the portal vein that caused Toll-like receptor (TLR) and NOD-like receptor (NLR) activation in the liver. TLR/NLR further activated the host-wide inflammatory response by inducing signaling cascades, such as the nuclear factor kappa B (NF-κB) transcriptional pathways, and accelerating the secretion of cytokines, such as tumor necrosis factor-alpha (TNF-α), which invokes chronic inflammation and leads to the development of liver lesions (Henao-Mejia et al., 2013; Schnabl, 2013; Chassaing et al., 2014; Schnabl and Brenner, 2014). According to Lu et al., the Bifidobacteriaceae/Enterobacteriaceae (B/E) ratio was significantly decreased in HBV-infected patients, indicating detractive microbial colonization resistance in the bowel (Lu et al., 2011). A species-level study performed by Xu et al. showed that within the same *Bifidobacterium* genus, species with potential benefits were significantly reduced in HBV patients, while opportunistically pathogenic species were significantly increased (Xu et al., 2012). Studies on patients with liver fibrosis and cirrhosis have further linked dysbiosis (Chen et al., 2011; Bajaj et al., 2014) and dysfunction (Qin et al., 2014) of the gut microbiota with severe damage and functional disorder in liver tissue, resulting in enhanced microbial functions involved in membrane transport and diminished microbial functions involved in carbohydrate metabolism, amino acid metabolism, energy metabolism, signal transduction and the metabolism of cofactors and vitamins (Qin et al., 2014). By rectifying the composition of the gut microbiota via prebiotic therapy, the endotoxemia in hepatitis B and C patients could be reduced (Chen et al., 2007). Meanwhile, the abnormal change in serum metabolites was also closely associated with liver disease. Unbalanced amino acid metabolism, especially high serum levels of aromatic amino acids (AAAs), including phenylalanine, tyrosine and tryptophan, is a major phenomenon indicating functional disorder in liver tissue and is critical in the pathogenesis of chronic liver diseases, including liver fibrosis, cirrhosis and HCC (Morgan et al., 1978; Heberer et al., 1980; Dejong et al., 2007; Gao et al., 2015; Sands et al., 2015). A lower ratio of branched-chain amino acids to AAAs is considered to play a causal role in liver failure (Morgan et al., 1978; Dejong et al., 2007). A recent study also noted serum metabolite abnormalities in liver disease, such as increased phenylalanine, malic acid and 5-methoxytryptamine in HBV patients vs. healthy controls, increased palmitic acid in patients with cirrhosis vs. HBV, and increased asparagine and β-glutamate in patients with HCC vs. cirrhosis (Gao et al., 2015). As previous evidence has revealed that the gut microbiota modulates the hosts' metabolic phenotypes (Claus et al., 2008; Li et al., 2008), it is of obvious value to determine whether and how the dysbiosis and dysfunction of the gut microbiota is linked with such changes in serum metabolites in the context of liver disease. Hence, elucidating the characteristics of the gut microbiota in CHB patients will help unravel the detailed pathogenic role of gut dysbiosis in the progression of liver disease and guide the management of gut microbiota-targeted therapies.

To fulfill the above research purposes, our study focused on early-stage CHB patients whose Child-Pugh scores were not >9. These patients were selected due to the clinical consideration that they had not experienced severe liver damage or metabolic disorder. The altered metabolites of patients with higher Child-Pugh scores could result from dysfunction of the microbiota, but in the meantime, they could also affect the composition of the gut microbiota. Our aim was to determine whether the gut microbiota is already altered at the beginning of liver damage and discover its potential pathogenic role. To date, previous studies on CHB patients using quantitative PCR to examine 16S rRNA genes have been limited to investigating changes in the abundance of specific genera or species of interest (Lu et al., 2011; Xu et al., 2012). In this study, we will utilize the Illumina MiSeq high-throughput sequencing platform to profile a compositional and functional overview of the gut microbiota in CHB patients. The association between the gut microbiota and hosts' physiological indices and serum metabolome will be further investigated.

## Materials and methods

### Patients

All subjects enrolled had physical examinations performed, as previously described (Kang et al., 2015; Lu et al., 2015) in three hospitals, the Shuguang Hospital (the affiliated hospital of Shanghai University of Traditional Chinese Medicine), the Infectious Disease Hospital of Ningbo and the Sixth of People's Hospital of Shaoxing Zhejiang, People's Republic of China. In brief, subjects who had received antibiotic treatment within 1 month before enrollment were excluded, along with patients with liver cirrhosis, hepatocellular carcinoma (HCC) or other diseases. A total of 107 subjects were finally selected for inclusion in this study, including 85 CHB patients and 22 healthy volunteers matched in age, gender and body mass index (BMI). The CHB patients were diagnosed based on increased alanine aminotransferase (ALT) levels (above the upper limit of the normal range, 0–40 U/L) in at least two blood samples assayed over a 6-month period and the presence of detectable hepatitis B surface antigen and/or HBV DNA.

### Clinical trial number

This project was approved by the Chinese Clinical Trial Registry (Registration Number: ChiCTR-DCC-10000759) and the ethics committee and IRB of the Shuguang Hospital affiliated with the Shanghai University of TCM (Permit Number: 2012-206-22-01). The study was performed in accordance with the approved guidelines. All participants provided informed consent, and the study protocol conformed to the ethical guidelines of the Declaration of Helsinki (2008).

### Physiological indices assessment

Serum levels of total bilirubin (TBIL), direct bilirubin (DBIL), indirect bilirubin (IDBIL), alanine aminotransferase (ALT), aspartate aminotransferase (AST), gamma glutamyl transferase (GGT), and albumin (ALB) were measured by an automatic biochemical analyzer (Model LX-20; Beckman, Fullerton, USA).

### Stool sample collection and microbial DNA extraction

Stool samples were collected on the day of the medical examination and immediately frozen at −80°C. DNA was extracted from fecal samples and purified using the Omega Gel Extraction kit (D2501-01, OMEGA Bio-Tek, Taiwan, China).

### Sequencing procedures

Hypervariable region V3-V4 amplicons from the 16S rRNA gene were sequenced by Illumina MiSeq 2 × 300 bp paired-end sequencing, as described (https://support.illumina.com/documents/documentation/chemistry_documentation/16s/16smetagenomic-library-prep-guide-15044223-b.pdf), with the following modifications: Platinum Pfx DNA polymerase (C11708021, Invitrogen, USA) was used during amplification. The number of PCR cycles for the amplicon PCR (amplification of 16S rRNA V3-V4 region) was reduced to 21 to diminish PCR bias. The index PCR and PCR product purification were completed according to the protocol. The primers used for amplicon PCR were S-D-Bact-0341-b-S-17, 5′-CCTACGGGNGGCWGCAG-3′ and S-D-Bact-0785-a-A-21, 5′-GACTACHVGGGTATCTAATCC-3′ (Klindworth et al., 2013).

### Accession codes

The 16S rRNA V3-V4 amplicon sequencing data are available in the NCBI Short Read Archive repository under BioProject PRJNA382861 (http://www.ncbi.nlm.nih.gov/bioproject/PRJNA382861). Detailed SRA accession number and the corresponding metadata of each sample are listed in Table S1.

### Clustering sequences into operational taxonomic units (OTUs)

Sequence merging, error correction and quality control were performed using moira v1.1.0 (Puente-Sánchez et al., 2016). The PCR primers were subsequently truncated. The sequence lengths were restricted to >400 nt. Sequences were de-replicated into unique sequences and aligned with the SILVA bacteria reference database (Quast et al., 2013) with the “align.seqs” command in mothur (Schloss et al., 2009). The alignment space was optimized by removing the sequences that failed to align correctly to ensure that all the remaining sequences overlapped at the same region of the SILVA Reference Alignment. The aligned unique sequences were divided by samples and checked for chimeras using abundant sequences as references with the UCHIME (Edgar et al., 2011) *de novo* algorithm. The non-chimeric sequences were then classified according to the mothur-formatted version of the RDP classifier training set v9 (Cole et al., 2014), and the sequences failed to be classified as bacteria were further filtered out. The final qualified sequences were rarefied to 10,000 per sample to minimize the bias due to unbalanced sequencing depth. The qualified unique sequences were sorted in descending order of abundance, and the singletons were set aside. Non-chimeric OTU representative sequences were selected with 97% similarity threshold by UPARSE (Edgar, 2013). The OTU table was finalized by mapping all unique sequences to the obtained OTUs with the USEARCH (Edgar, 2010) global alignment algorithm.

### Definition of the gut dysbiosis index (GDI)

We propose a GDI to measure the severity of the overall gut microbiota shift in patients with a specified disease compared with healthy controls, which is expressed by Equation (1):

(1) $$\begin{array}{r} \text{GDI }=\;\frac{\sum {\text{OTU}}_{p}}{P}-\frac{\sum {\text{OTU}}_{h}}{H} \end{array}$$

wherein OTU_p_ represents the patient-enriched OTUs and OTU_h_ represents the healthy-enriched OTUs identified by LEfSe (Segata et al., 2011). ∑OTU_p_ is the summed abundance of OTU_p_, and ∑OTU_h_ is the summed abundance of OTU_h_; *P* and *H* represent the number of OTUs belonging to OTU_p_ and OTU_h_, respectively. A larger GDI value indicates a more severe gut dysbiosis status.

### Inference of metagenomic functional contents of the gut microbiota

The qualified 16S rRNA sequences were aligned against Greengenes (DeSantis et al., 2006) pre-defined 97%-level OTU database for annotation. To minimize the influence of sequencing errors, initially non-singleton sequences were aligned to the Greengenes database, and then the reference sequences that were matched at least once were obtained to form a new database. Closed-reference OTU picking was performed in this newly defined database using the USEARCH (Edgar, 2010) global alignment algorithm. The OTU table was normalized by the sequencing depth, and the corresponding functional genes were predicted by PICRUSt v1.0.0 (Langille et al., 2013). Finally, the predicted genes were mapped onto Kyoto Encyclopedia of Genes and Genomes (KEGG) (Kanehisa et al., 2008) level-2 and level-3 pathways using HUMAnN v0.99 (Abubucker et al., 2012).

### Serum sample preparation

Protein precipitation and metabolite extraction were conducted as previously described (Dai et al., 2013). Each 100-μL serum sample was combined with 10 μL of heptadecanoic acid (1 mg/mL) and 300 μL of solvent (methanol: chloroform, 3:1, V/V). After vortex-mixing for 30 s, the samples were conditioned at −20°C for 10 min and centrifuged at 10,000 rpm for 10 min. A 200-μL aliquot of the supernatant was then transferred into a gas chromatography (GC) vial and evaporated to dryness under N_2_ at 30°C. By adding 80 μL of methoxyamine in pyridine (15 mg/mL) to the GC vial and vortex-mixing for 30 s, the methoximation reaction was carried out for 90 min under rocking in a shaker at 30°C. Then, trimethylsilylation was performed for another 1 h at 70°C through adding 80 μL of N,O-bis(trimethylsilyl) trifluoroacetamide plus 1% trimethylchlorosilane to the samples. Finally, the solution was vortexed for 30 s and ready for gas chromatography mass spectrometry (GC-MS) measurement.

### Serum metabolome measurement

All GC-MS analyses were performed by a 5975B mass spectrometer (Agilent technologies, USA) coupled to an Agilent 6890 (Agilent technologies, USA) GC instrument using a capillary column (Agilent J&W DB-5ms Ultra Inert 30 m × 0.25 mm, film thickness 0.25 μm). Helium carrier gas was used at a constant flow rate of 1.0 mL/min. One microliter of each derivatized sample was injected into the GC-MS instrument using the splitless injection mode. A column temperature program, as listed in Table S2, was optimized to acquire good separation. The injection port, interface and source temperature was set at 270°, 260°, and 220°C, respectively. The measurements were made with electron impact ionization (70 eV) in the full scan mode (m/z 30–550). The solvent post time was set to 5 min. The GC-MS operating conditions were the same as those in a previous experiment (Sun et al., 2012) except the column temperature program. Metabolites were then identified by searching in the NIST 2011 database and verified by standards.

### Statistical analysis

Matched samples from the CHB patients and healthy control subjects were selected using the MatchIt (Ho et al., 2011) package in R (R Core Development Team and R Core Team, 2015). The permutational multivariate analysis of variance (PERMANOVA) test and distance-based redundancy analysis were performed using the vegan (Oksanen et al., 2016) package in R. The linear discriminant analysis (LDA) effect size (LEfSe) method was implemented by LEfSe v1.0 (Segata et al., 2011). Receiver operating characteristic (ROC) curves were calculated using the pROC (Robin et al., 2011) package in R. Sparse partial least squares (sPLS) analysis was performed using the mixOmics v6.1.0 (Le Cao et al., 2016) R package. The *p*-values of multiple comparisons were post-adjusted using the false discovery rate approach (Storey, 2003).

## Results

### Physiological characteristics of patients

One hundred and seven subjects (22 healthy subjects and 85 CHB patients) were enrolled in this study. The physiological characteristics of the cohort are shown in Table 1. The Child-Pugh score (Child and Turcotte, 1964; Pugh et al., 1973) was calculated from 5 criteria (TBIL, serum ALB, prothrombin time, ascites and hepatic encephalopathy) to indicate liver disease severity. According to the Child-Pugh score, 76 out of 85 CHB patients were classified as phase A (score = 5~6), i.e., having the least severe liver disease. The other 9 patients were classified as phase B (score = 7~9), i.e., having moderately severe liver disease. The CHB patients had significantly higher ALT, AST, and GGT levels, and the phase B patients had significantly decreased ALB and increased DBIL, IDBIL and TBIL levels.

**Table 1:** Physiological characteristics of the subjects.

	**Healthy**	**CHB**		***p*-value**
		**PhaseA**	**PhaseB**	
Sample size	22	76	9	–
Age	36 (28, 45)	38 (30, 43)	37 (33, 39)	0.95
Gender, male/female	13/9	49/27	6/3	0.94
BMI (kg/m^2^)	22 (21, 24)	22 (20, 24)	21 (20, 22)	0.81
ALT (IU/L)	17 (15, 21)	44 (26, 71)	126 (31, 384)	<0.01
AST (IU/L)	19 (16, 20)	35 (27, 59)	107 (44, 203)	<0.01
GGT (IU/L)	20 (18, 22)	27 (17, 45)	136 (48, 265)	0.01
ALB (g/L)	43 (40, 45)	45 (43, 47)	34 (33, 41)	<0.01
TBIL (μmol/L)	15 (12, 17)	16 (13, 20)	39 (25, 51)	<0.01
DBIL (μmol/L)	4 (3, 4)	4 (3, 6)	20 (10, 29)	<0.01
IDBIL (μmol/L)	11 (8, 13)	11 (9, 14)	22 (16, 25)	<0.01

*Quantitative results are expressed as median with first and third quartiles into brackets*.

*P values indicate the significance of difference among groups*.

*P values were adjusted with FDR method*.

*AST, aspartate aminotransferase; ALT, alanine aminotransferase; GGT, gamma-glutamyl transpeptidase; ALB, albumine; BA, bile acid; TBIL, total bilirubin; DBIL, direct bilirubin; IDBIL, indirect bilirubin*.

### Overview of gut microbial shift in patients

After paired-end merging, error correction and quality filtration, the qualified sequences were rarefied to 10,000 sequences per sample and clustered into 886 *de novo* OTUs at the 97% similarity threshold level.

**Figure 1:**
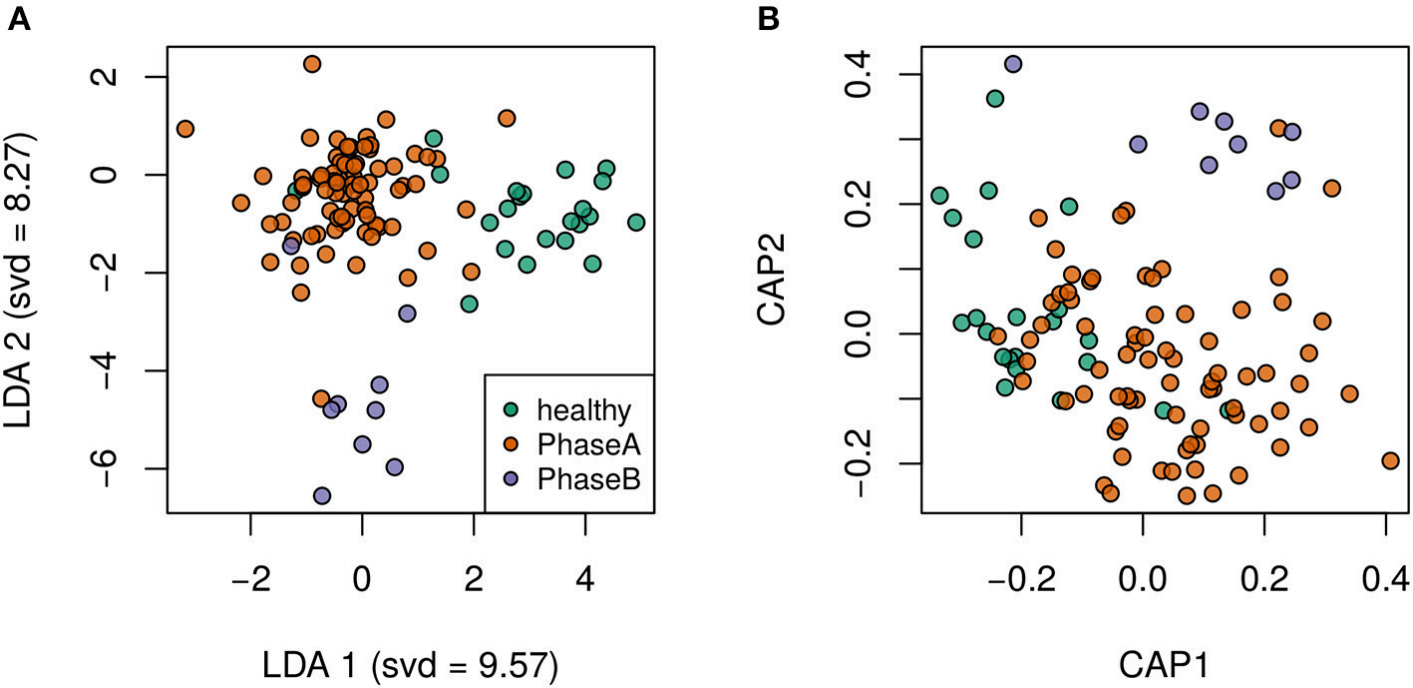
Clear separation by health status was observed via supervised discriminant analyses. **(A)** Linear discriminant analysis plot. **(B)** Bray-Curtis distance-based redundancy discriminant analysis plot.

Principal component analysis plots based on the OTU distributions showed that the overall compositions of the gut microbiota were not significantly shifted by age (*p* = 0.09), gender (*p* = 0.99), BMI (*p* = 0.30) or antiviral treatment (*p* = 0.11) (PERMANOVA test, Figure S1). CHB itself was the primary driver of alterations to the gut microbiota in this study. Both the LDA plot based on the OTU distributions (Figure 1A) and the Bray-Curtis distance-based redundancy analysis plot (Figure 1B) showed a clear separation between the diseased and healthy groups (*p* < 0.01, PERMANOVA test). Within the CHB group, the gut microbiota was not significantly different between Child-Pugh phases A and B (*p* = 0.19, PERMANOVA test). The alpha diversities, including the OTU richness and phylogenetic diversities, were not significantly different between the patients and healthy subjects (Figure S2).

We used LEfSe (Segata et al., 2011) to identify the differential OTUs by comparing the CHB patients with the healthy controls. A total of 70 OTUs were detected as significantly increased or decreased (log(LDA) > 2, *p* < 0.01); of these, 32 OTUs were distributed prevalently in more than 10% of the samples (Table 2). Five OTUs were enriched in the CHB patients, while the other 27 OTUs were enriched in the healthy controls. Notably, under the same family of Lachnospiraceae, OTU270 was enriched in CHB, while OTU418, OTU 877, and OTU111 were depleted.

**Table 2:** Differential OTUs selected by linear discriminant analysis effect size (LEfSe).

**OTUID**	**Family**	**Genus**	**Enriched class**	**LDA**	***p*-value**
OTU673	Actinomycetaceae	Actinomyces	CHB	2.82	0.01
OTU158	Clostridiaceae	Clostridium sensu stricto	CHB	2.40	0.05
OTU270	Lachnospiraceae	Unclassified Lachnospiraceae	CHB	3.09	0.03
OTU17	Veillonellaceae	Megamonas	CHB	3.68	*p* < 0.01
OTU829	Veillonellaceae	Megamonas	CHB	3.28	*p* < 0.01
OTU7	Bacteroidaceae	Bacteroides	Healthy	4.15	*p* < 0.01
OTU156	Bacteroidaceae	Bacteroides	Healthy	2.43	0.02
OTU35	Bacteroidaceae	Bacteroides	Healthy	3.71	0.03
OTU260	Coriobacteriaceae	Asaccharobacter	Healthy	2.49	0.04
OTU323	Coriobacteriaceae	Unclassified Coriobacteriaceae	Healthy	2.52	0.04
OTU10	Enterobacteriaceae	Escherichia/Shigella	Healthy	4.11	0.04
OTU164	Enterobacteriaceae	Unclassified Enterobacteriaceae	Healthy	3.02	*p* < 0.01
OTU418	Lachnospiraceae	Unclassified Lachnospiraceae	Healthy	2.88	0.02
OTU877	Lachnospiraceae	Unclassified Lachnospiraceae	Healthy	2.90	*p* < 0.01
OTU111	Lachnospiraceae	Unclassified Lachnospiraceae	Healthy	3.01	0.01
OTU149	Porphyromonadaceae	Butyricimonas	Healthy	2.60	*p* < 0.01
OTU16	Porphyromonadaceae	Parabacteroides	Healthy	3.46	0.04
OTU48	Porphyromonadaceae	Parabacteroides	Healthy	3.44	*p* < 0.01
OTU150	Rikenellaceae	Alistipes	Healthy	2.14	0.02
OTU11	Rikenellaceae	Alistipes	Healthy	3.84	0.03
OTU96	Rikenellaceae	Alistipes	Healthy	2.74	0.01
OTU103	Ruminococcaceae	Clostridium IV	Healthy	3.00	0.01
OTU147	Ruminococcaceae	Ruminococcus	Healthy	2.87	0.03
OTU108	Ruminococcaceae	Unclassified Ruminococcaceae	Healthy	2.96	0.03
OTU51	Ruminococcaceae	Unclassified Ruminococcaceae	Healthy	3.16	0.04
OTU115	UnclassifiedBacteria	Unclassified Bacteria	Healthy	2.81	0.03
OTU66	UnclassifiedClostridiales	Unclassified Clostridiales	Healthy	3.02	*p* < 0.01
OTU176	UnclassifiedClostridiales	Unclassified Clostridiales	Healthy	2.32	0.01
OTU229	UnclassifiedClostridiales	Unclassified Clostridiales	Healthy	2.13	0.01
OTU293	UnclassifiedClostridiales	Unclassified Clostridiales	Healthy	2.03	0.03
OTU74	UnclassifiedClostridiales	Unclassified Clostridiales	Healthy	2.47	0.01
OTU90	UnclassifiedClostridiales	Unclassified Clostridiales	Healthy	2.70	0.02

### Diagnosis of CHB using the GDI

Based on the 32 differential OTUs identified above, the GDI was introduced to measure the severity of the overall gut microbiota shift in patients with respect to the healthy controls. The GDIs were −6.82 (−25.14, 20.14) (median, first quartile, third quartile) in patients and −60.25 (−89.05, −35.25) in the healthy controls. The CHB group had significantly higher GDI values (*p* < 0.01, Wilcoxon test), showing a more severe ratio of “bad vs. good” taxa abundance associated with liver disease.

**Figure 2:**
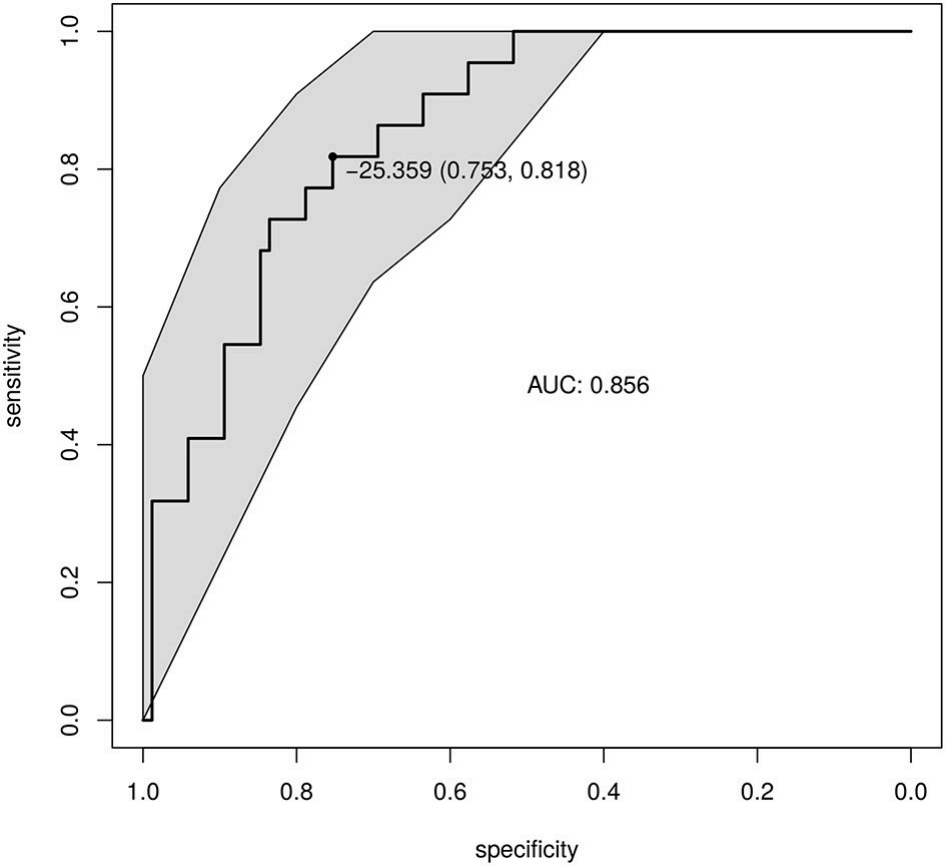
Diagnosis of CHB based on the GDI. The GDI was calculated based on the differential and prevalent OTUs. The ROC of the GDI produced by leave-one-out cross-validation is shown. The point shows the GDI threshold chosen based on Youden's J statistic, and the corresponding specificity and sensitivity.

ROC curves were built to define a diagnostic threshold of CHB based on the GDI values (Figure 2). The leave-one-out cross-validation of the area under the ROC curves achieved 0.86 (95% confidence interval: 0.78–0.93), showing promising diagnostic potential. The optimal GDI threshold was then chosen at the ROC cut-off that maximized the Youden's J statistic (Youden, 1950). The GDI threshold was set to −25.36, and the corresponding accuracy, sensitivity and specificity were 0.77, 0.75, and 0.81.

### Inferred metagenomic function of the gut microbiota

By mapping sequences to the Greengenes database (DeSantis et al., 2006), the functional gene contents of the gut microbiota were predicted by PICRUSt (Langille et al., 2013) and were mapped on KEGG (Kanehisa et al., 2008) orthologues by HUMAnN (Abubucker et al., 2012). The KEGG orthologues (ko) enriched or depleted in patients were assessed by LEfSe (Segata et al., 2011; Table 3).

**Table 3:** Functional orthologues of gut microbiota enriched in different disease status.

**KEGG level2**	**KEGG level3**	**Enriched class**	**LDA**	***p*-value**
**Amino acid metabolism**		Healthy	3.66	*p* < 0.01
Amino acid metabolism	Arginine and proline metabolism ko00330	Healthy	2.97	*p* < 0.01
Amino acid metabolism	Glycine serine and threonine metabolism ko00260	CHB	3.15	*p* < 0.01
Amino acid metabolism	Histidine metabolism ko00340	Healthy	3.47	*p* < 0.01
Amino acid metabolism	Lysine degradation ko00310	Healthy	2.82	*p* < 0.01
Amino acid metabolism	Phenylalanine metabolism ko00360	Healthy	2.79	*p* < 0.01
Amino acid metabolism	Tryptophan metabolism ko00380	Healthy	2.85	*p* < 0.01
**Carbohydrate metabolism**		Healthy	3.43	*p* < 0.01
Carbohydrate metabolism	Amino sugar and nucleotide sugar metabolism ko00520	Healthy	2.76	0.03
Carbohydrate metabolism	Fructose and mannose metabolism ko00051	Healthy	2.87	0.02
Carbohydrate metabolism	Inositol phosphate metabolism ko00562	Healthy	2.68	*p* < 0.01
Carbohydrate metabolism	Pentose phosphate pathway ko00030	CHB	2.70	0.02
Carbohydrate metabolism	Pyruvate metabolism ko00620	Healthy	2.78	0.03
Carbohydrate metabolism	Starch and sucrose metabolism ko00500	Healthy	3.00	0.01
**Cell growth and death**		CHB	3.25	*p* < 0.01
Cell growth and death	Cell cycle Caulobacter ko04112	CHB	3.25	*p* < 0.01
**Energy metabolism**		CHB	3.14	*p* < 0.01
Energy metabolism	Oxidative phosphorylation ko00190	CHB	3.09	*p* < 0.01
**Glycan biosynthesis and metabolism**		–	–	–
Glycan biosynthesis and metabolism	Glycosaminoglycan degradation ko00531	Healthy	3.56	0.03
Glycan biosynthesis and metabolism	Peptidoglycan biosynthesis ko00550	CHB	2.86	*p* < 0.01
**Infectious diseases**		CHB	2.74	*p* < 0.01
Infectious diseases	Vibrio cholerae pathogenic cycle ko05111	CHB	2.74	*p* < 0.01
**Lipid metabolism**		–	–	–
Lipid metabolism	Fatty acid biosynthesis ko00061	CHB	3.16	0.02
**Membrane transport**		CHB	3.10	*p* < 0.01
Membrane transport	ABC transporters ko02010	CHB	2.95	*p* < 0.01
Membrane transport	Phosphotransferase system PTS ko02060	CHB	2.71	0.01
**Metabolism of cofactors and vitamins**		–	–	–
Metabolism of cofactors and vitamins	Biotin metabolism ko00780	Healthy	3.24	0.02
Metabolism of cofactors and vitamins	Folate biosynthesis ko00790	Healthy	2.90	0.02
Metabolism of cofactors and vitamins	Lipoic acid metabolism ko00785	Healthy	3.69	*p* < 0.01
Metabolism of cofactors and vitamins	Nicotinate and nicotinamide metabolism ko00760	CHB	3.26	*p* < 0.01
Metabolism of cofactors and vitamins	One carbon pool by folate ko00670	CHB	2.93	*p* < 0.01
Metabolism of cofactors and vitamins	Pantothenate and CoA biosynthesis ko00770	CHB	3.10	*p* < 0.01
Metabolism of cofactors and vitamins	Porphyrin and chlorophyll metabolism ko00860	CHB	3.18	*p* < 0.01
Metabolism of cofactors and vitamins	Riboflavin metabolism ko00740	CHB	3.52	*p* < 0.01
Metabolism of cofactors and vitamins	Thiamine metabolism ko00730	CHB	2.96	0.01
**Metabolism of other amino acids**		–	–	–
Metabolism of other amino acids	Phosphonate and phosphinate metabolism ko00440	Healthy	2.83	0.01
**Metabolism of terpenoids and polyketides**		CHB	2.81	*p* < 0.01
Metabolism of terpenoids and polyketides	Terpenoid backbone biosynthesis ko00900	CHB	2.81	*p* < 0.01
**Nucleotide metabolism**		CHB	3.15	*p* < 0.01
Nucleotide metabolism	Purine metabolism ko00230	CHB	2.88	*p* < 0.01
Nucleotide metabolism	Pyrimidine metabolism ko00240	CHB	3.06	*p* < 0.01
**Replication and repair**		CHB	3.38	0.01
Replication and repair	Homologous recombination ko03440	CHB	2.85	0.02
Replication and repair	Mismatch repair ko03430	CHB	3.18	*p* < 0.01
**Translation**		CHB	2.85	*p* < 0.01
Translation	Ribosome ko03010	CHB	2.84	*p* < 0.01
Translation	RNA transport ko03013	CHB	2.91	0.02
**Transport and catabolism**		Healthy	2.48	*p* < 0.01
Transport and catabolism	Peroxisome ko04146	Healthy	2.48	*p* < 0.01
**Xenobiotics biodegradation and metabolism**		Healthy	2.57	0.05
Xenobiotics biodegradation and metabolism	Naphthalene degradation ko00626	Healthy	2.57	0.05

The healthy controls showed 17 enriched KEGG level-3 functional orthologues, which were related to Amino Acid Metabolism (arginine and proline metabolism ko00330, histidine metabolism ko00340, lysine degradation ko00310, phenylalanine metabolism ko00360, tryptophan metabolism ko00380), Carbohydrate Metabolism (amino sugar and nucleotide sugar metabolism ko00520, fructose and mannose metabolism ko00051, inositol phosphate metabolism ko00562, pyruvate metabolism ko00620, starch and sucrose metabolism ko00500), Glycan Biosynthesis and Metabolism (glycosaminoglycan degradation ko00531), Metabolism of Cofactors and Vitamins (biotin metabolism ko00780, folate biosynthesis ko00790, lipoic acid metabolism ko00785), Metabolism of Other Amino Acids (phosphonate and phosphinate metabolism ko00440), Transport and Catabolism (peroxisome ko04146), and Xenobiotics Biodegradation and Metabolism (naphthalene degradation ko00626).

The CHB patients showed 21 enriched level-3 functional orthologues, which were related to Amino Acid Metabolism (glycine serine and threonine metabolism ko00260), Carbohydrate Metabolism (pentose phosphate pathway ko00030), Cell Growth and Death (cell cycle caulobacter ko04112), Energy Metabolism (oxidative phosphorylation ko00190), Glycan Biosynthesis and Metabolism (peptidoglycan biosynthesis ko00550), Infectious Diseases (vibrio cholerae pathogenic cycle ko05111), Lipid Metabolism (fatty acid biosynthesis ko00061), Membrane Transport (ABC transporters ko02010, phosphotransferase system PTS ko02060), Metabolism of Cofactors and Vitamins (nicotinate and nicotinamide metabolism ko00760, one carbon pool by folate ko00670, pantothenate and CoA biosynthesis ko00770, porphyrin and chlorophyll metabolism ko00860, riboflavin metabolism ko00740, thiamine metabolism ko00730), Metabolism of Terpenoids and Polyketides (terpenoid backbone biosynthesis ko00900), Nucleotide Metabolism (purine metabolism ko00230, pyrimidine metabolism ko00240), Replication and Repair (homologous recombination ko03440, mismatch repair ko03430), and Translation (ribosome ko03010, RNA transport ko03013).

### OTUs associated with the clinical indices

**Figure 3:**
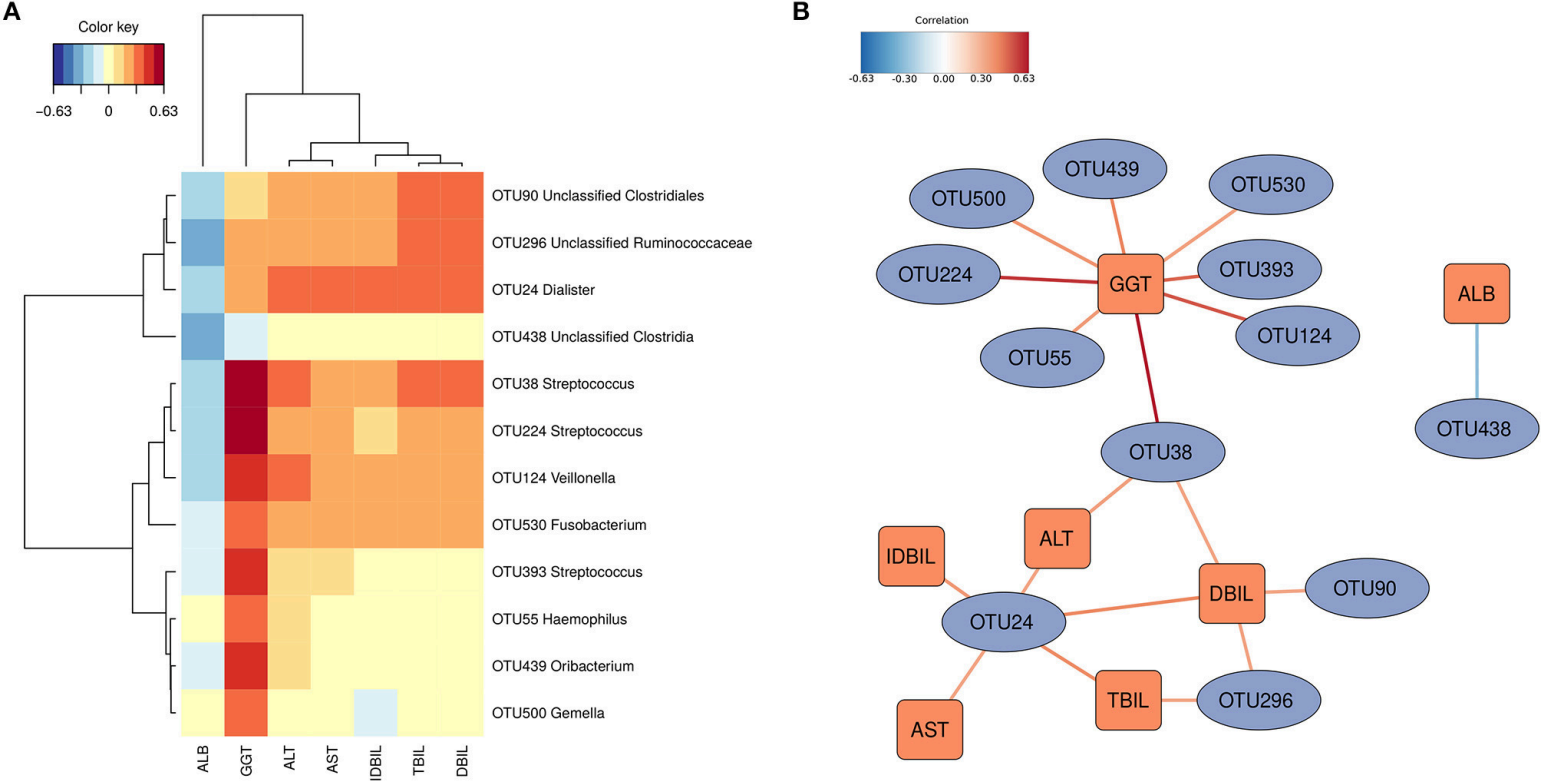
OTUs associated with hosts' clinical indices. Significant correlations were detected by sPLS regression (|correlation r| > 0.3). **(A)** Heatmap based on the correlation coefficients. **(B)** Relevant association network based on the sPLS regression. The OTUs mainly formed two clusters.

sPLS regression was performed to link the gut microbiota and the clinical indices. Strong correlations (|correlation r| > 0.3) were detected among 12 OTUs and the 7 physiological indices (Figure 3A). The 12 OTUs mainly formed two clusters (Figure 3B). One cluster was positively associated with TBIL, DBIL, IDBIL, AST and ALT. It contained 3 OTUs belonging to *Dialister*, unclassified Clostridiales and unclassified Ruminococcaceae. Another cluster showed a consistently strong association with GGT. It had 7 OTUs belonging to *Streptococcus, Veillonella, Fusobacterium, Haemophilus, Oribacterium*, and *Gemella*. These two clusters were connected via OTU38, which belonged to *Streptococcus*. Only OTU438 (unclassified Clostridia) was significantly negatively associated with ALB.

### OTUs associated with the serum metabolome

**Figure 4:**
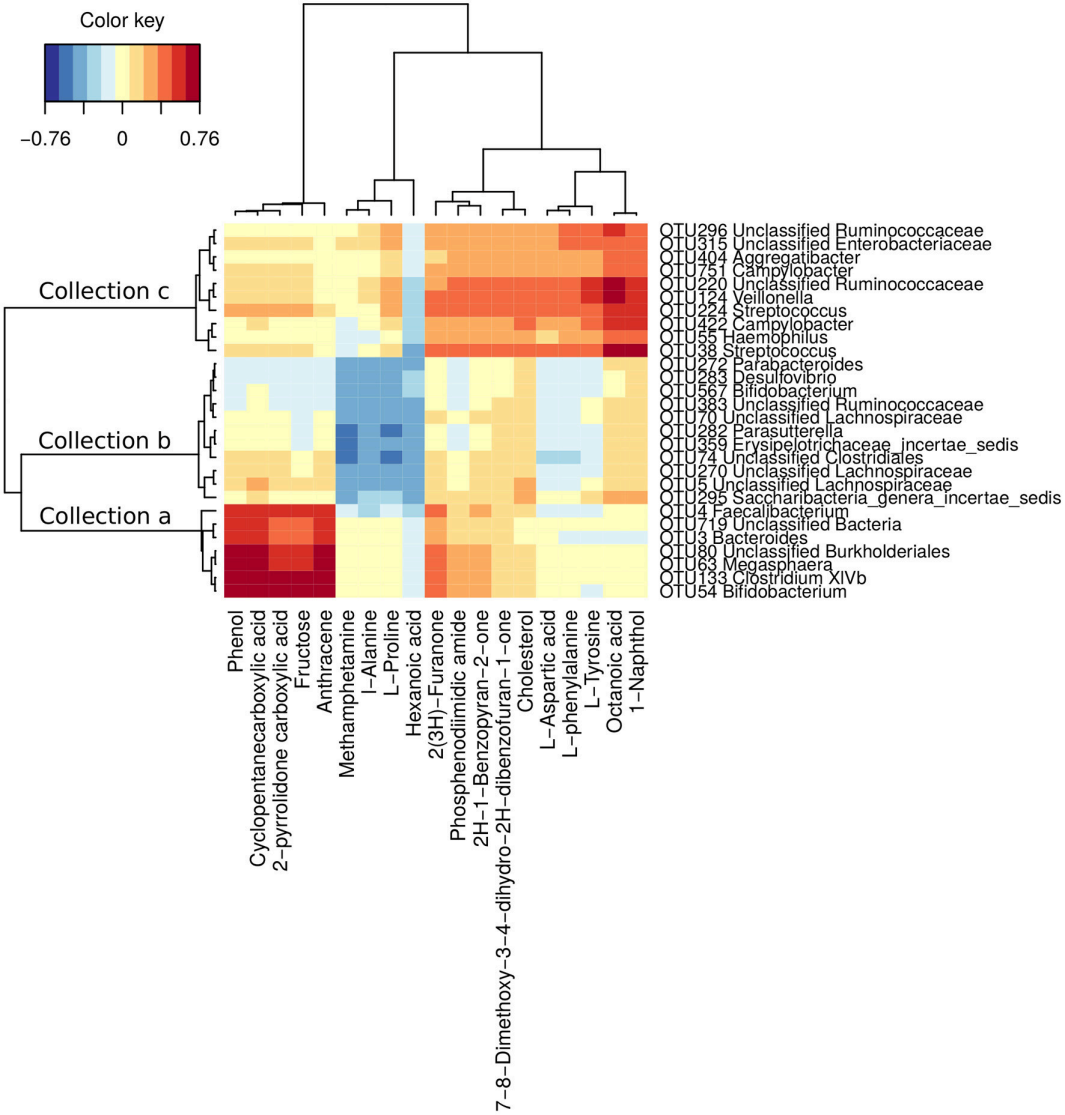
OTUs associated with hosts' serum metabolites. Correlations were detected by sPLS regression analysis (|correlation r| > 0.4). The OTUs were clustered into 3 collections by Ward's clustering.

To further understand the potential influence of gut dysbiosis on hosts, the serum metabolome of 40 subjects was measured (29 CHB patients and 11 healthy controls, Table S3). The sPLS regression analysis identified close relationship between 28 OTUs of the gut microbiota and 19 serum metabolites in hosts (|correlation r| > 0.4). The 28 OTUs were clustered into 3 collections by Ward's clustering (collections a, b and c, Figure 4). Collection a was positively associated with phenol, cyclopentanecarboxylic acid, 2-pyrrolidone carboxylic acid, fructose and anthracene. It contained 7 OTUs belonging to *Bifidobacterium, Clostridium XIVb, Megasphaera, Bacteroides, Faecalibacterium* and unclassified genera of Burkholderiales and Bacteria. Collection b was negatively associated with methamphetamine, L-alanine, L-proline and hexanoic acid. It included 11 OTUs belonging to *Saccharibacteria genera incertae sedis, Erysipelotrichaceae incertae sedis, Parasutterella, Bifidobacterium, Desulfovibrio, Parabacteroides* and unclassified genera of Lachnospiraceae, Clostridiales and Ruminococcaceae. Collection c was positively associated with 2(3H)-furanone, phosphenodiimidic amide, 2H-1-benzopyran-2-one, 7,8-dimethoxy-3,4-dihydro-2H-dibenzofuran-1-one, cholesterol, L-aspartic acid, L-phenylalanine, L-tyrosine, octanoic acid and 1-naphthol. This collection had 10 OTUs belonging to *Streptococcus, Haemophilus, Campylobacter, Veillonella, Aggregatibacter*, and unclassified genera of Ruminococcaceae and Enterobacteriaceae.

By accumulating the abundances of OTUs in the same collection, collection c was significantly more abundant in phase B than in phase A patients (*p* < 0.01, Wilcoxon test), while collections a and b were not (p values were 0.62 and 0.87, respectively, Wilcoxon test) (Figure S3). The multivariate PERMANOVA test showed similar results. The OTUs in collection c were significantly (*p* = 0.03) differently distributed between phase A and B patients, while the OTUs in the other collections were not (*p*-values were 0.88 and 0.16, respectively).

Particularly, four OTUs, OTU38 (*Streptococcus*), OTU124 (*Veillonella*), OTU224 (*Streptococcus*) and OTU55 (*Haemophilus*), were closely associated with both the clinical indices and the accumulation of 10 serum metabolites, especially L-phenylalanine and L-tyrosine, which are AAAs that play a pathogenic role in liver disease (Morgan et al., 1978; Heberer et al., 1980; Dejong et al., 2007; Gao et al., 2015; Sands et al., 2015). In the CHB patients, the abundances of these four OTUs were significantly higher in the Child-Pugh phase B group (Figure S4, *p*-values were 0.03, < 0.01, 0.01, and 0.02, respectively, Wilcoxon test).

## Discussion

Along with previous evidence, the current findings indicate that the gut microbiota should be an unignorably influential factor of CHB progression to severe liver failure. In this study, the observed compositional and functional shifts in the gut microbiota of CHB patients echo the findings of previous studies on patients with liver fibrosis and cirrhosis. At the family level, similar to Chen et al.'s and Bajaj et al.'s work (Chen et al., 2011; Bajaj et al., 2014), 2 OTUs increased in CHB belong to Veillonellaceae, while 13 OTUs decreased in CHB belong to Lachnospiraceae, Rikenellaceae, Porphyromonadaceae and Ruminococcaceae. At the genus level, 9 OTUs depleted in CHB belong to *Alistipes, Bacteroides, Parabacteroides* and *Ruminococcus*, which is similar to Qin et al.'s results (Qin et al., 2014). The alterations in such taxa were reported to result in the reduced production of short-chain fatty acids and antibacterial peptides, causing a detractive intestinal barrier against the colonization of pathogenic microbes in liver disease (Henao-Mejia et al., 2013; Schnabl, 2013; Chassaing et al., 2014; Schnabl and Brenner, 2014). At the inferred metagenomic pathway level, 2 KEGG orthologues enriched in CHB patients belong to Membrane Transport, while 10 KEGG orthologues enriched in healthy controls belong to Carbohydrate Metabolism and Amino Acid Metabolism, which is consistent with Qin et al.'s results (Qin et al., 2014). In addition, OTU38 (*Streptococcus*), OTU124 (*Veillonella*), OTU224 (*Streptococcus*), and OTU55 (*Haemophilus*) were closely connected with the hosts' physical indices and the accumulation of 10 serum metabolites, and their abundances were significantly higher in the Child-Pugh phase B group than in the phase A group. Our results indicate that the composition of the gut microbiota has already changed in patients with CHB before the occurrence of severe liver lesions and is associated with the alterations in liver functions and serum metabolites, suggesting that the shift in the gut microbiota plays a potentially pathogenic role in liver disease. These findings provide new insight into gut microbiota-targeted interventions to improve the prognosis of CHB.

We proposed the GDI in this study to comprehensively describe the proportional change in the abundances of “bad” vs. “good” bacteria by measuring systematic shifts in the gut microbiota. Previously, Lu et al. introduced a hepatitis B-specific B/E ratio based on quantitative PCR (Lu et al., 2011). However, in our study, as well as in Bajaj et al.'s work (Bajaj et al., 2014), Bifidobacteriaceae was not detected as a predominant taxon (>1%) in healthy controls through high-throughput sequencing. Moreover, Xu et al. showed that even under the acknowledged beneficial genus *Bifidobacterium*, some species were actually opportunistic pathogens and were enriched in CHB (Xu et al., 2012). Bajaj et al. also introduced a cirrhosis dysbiosis ratio, which compares autochthonous to non-autochthonous taxa. However, it faces the same issue by applying the ratio at the family level. Indeed, all bacteria under a taxon may encode different functions and may have significantly divergent associations with disease phenotypes. Therefore, special caution is required when using summed abundances at upper taxonomic levels to indicate structural alterations in the gut microbiota (Zhang and Zhao, 2016). Our GDI calculation based on differential OTUs provides better resolution to describe the key relevant changes in the gut microbiota. The success of the GDI in this pilot study suggests its potential as a substitutional/supplemental application for the surveillance of gut dysbiosis and the guidance of prebiotic/probiotic therapy in CHB. Due to the large variation in the gut microbiota among the population, more representative subjects are needed to obtain a generalized GDI value in the future.

Using high-throughput sequencing and multi-omics strategies, we further emphasized the potential role of the gut-liver axis in the progression of liver disease through the abnormal accumulation of AAAs in the serum (Morgan et al., 1978; Heberer et al., 1980; Dejong et al., 2007; Gao et al., 2015; Sands et al., 2015). Elevated AAA concentrations adversely affect cerebral functions and play a causal role in disturbed neurotransmission and subsequent hepatic encephalopathy (Dejong et al., 2007). In liver disease, the accumulation of phenylalanine and tyrosine in the plasma is not associated with impaired catabolism, but only with increased uptake and production of such essential amino acids (Heberer et al., 1980; Tessari et al., 2008). In this study, we observed that 10 OTUs, particularly OTU38 (*Streptococcus*), OTU124 (*Veillonella*), OTU224 (*Streptococcus*), and OTU55 (*Haemophilus*), were positively associated with the serum levels of L-phenylalanine and L-tyrosine, and the abundances of these OTUs were significantly increased in the Child-Pugh phase B group. Additionally, the inferred metagenomic functions of the gut microbiota were depleted in the metabolic pathway of AAAs in CHB patients. These results indicate that the gut microbiota may at least be partially involved in the abnormal accumulation of serum metabolites, thus potentially playing a role in the metabolic pathogenesis of liver disease (Figure S5). Additional work including more subjects, preferably with varying degrees of liver lesion severity, and complete physiological examinations and serum metabolome information, is required to further investigate and validate such associations.

In this study, by scanning compositional and functional changes in the gut microbiota of CHB patients, we observed cross-talk among gut dysbiosis, physiological indices and serum metabolites that are reportedly associated with fibrotic and cirrhotic liver lesions. Consequently, the present work provides evidence supporting the potential role of the gut microbiota in the processes that drive CHB to progress to severe forms of liver failure, including inflammation and pathogenic metabolite accumulation. These results not only expand our knowledge of the essential role of the gut microbiota in liver disease from a novel perspective but also might facilitate therapeutic strategies for monitoring/altering gut dysbiosis in CHB patients.

## Author contributions

JW, YFZ, LZ, YYZ, YH, and MZ designed the study; JW, XZ, JL, and QZ performed the gut microbial experiments; YW, JD, and SS performed the serum metabolic experiments; YZ, JP, and QF performed the clinical trail; JW and MZ performed the statistical analyses; JW and MZ wrote the paper.

### Conflict of interest statement

The authors declare that the research was conducted in the absence of any commercial or financial relationships that could be construed as a potential conflict of interest.
