# Supplementary material for: Gut Microbial Dysbiosis Is Associated with Altered Hepatic Functions and Serum Metabolites in Chronic Hepatitis B Patients
Source: Front Microbiol. 2017 Nov 13;8:2222. doi: 10.3389/fmicb.2017.02222 (PMC5693892; doi:10.3389/fmicb.2017.02222)
Supplement: Supplementary file 1 [file Image1.PDF]

1 Supplementary Figures

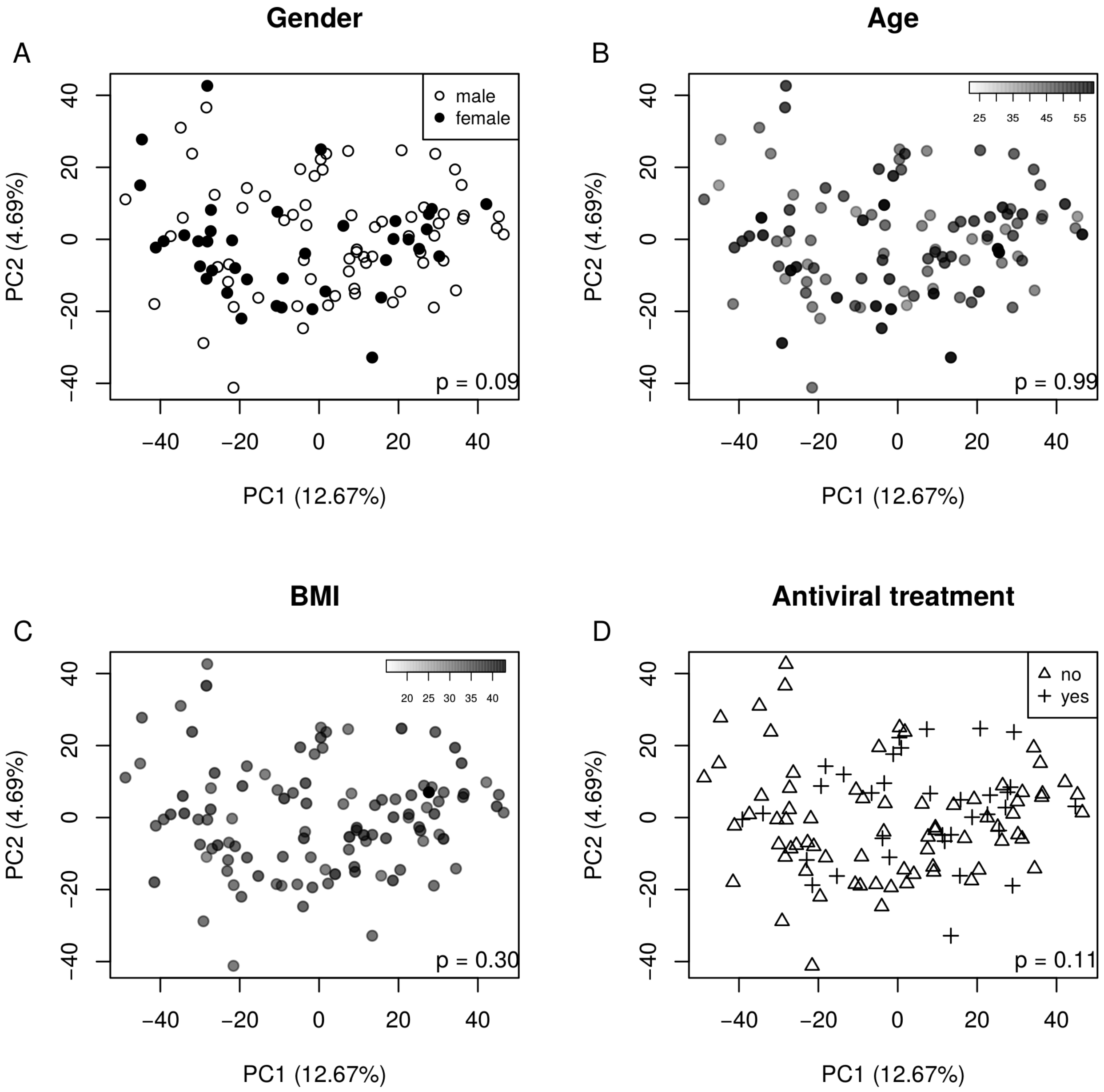

Supplementary Figure 1 Overall compositions of gut microbiota were not significantly affected by (A) gender, (B) age, (C) BMI or (D) the use of antiviral treatment. PCA plots were used to visualize the results. P values were calculated by the PERMANOVA test. BMI: body mass index. PCA: principal component analysis.
