# Supplementary material for: Gut Microbial Dysbiosis Is Associated with Altered Hepatic Functions and Serum Metabolites in Chronic Hepatitis B Patients
Source: Front Microbiol. 2017 Nov 13;8:2222. doi: 10.3389/fmicb.2017.02222 (PMC5693892; doi:10.3389/fmicb.2017.02222)
Supplement: Supplementary file 2 [file Image2.PDF]

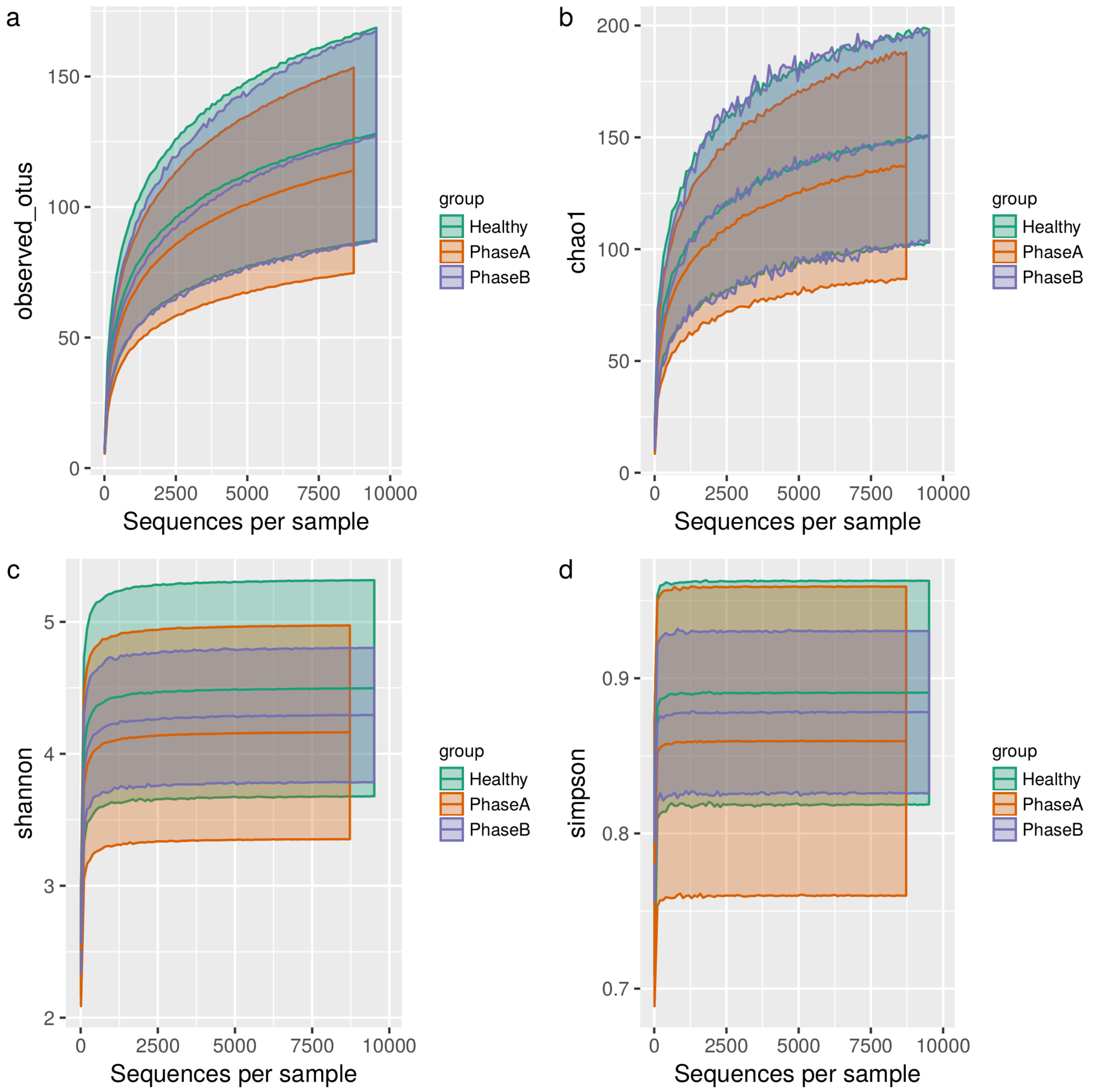

Supplementary Figure 2 The rarefaction curves of (a) number of OTUs and (b) Chao1, (c) Shannon and (d) Simpson indices observed in each sample. There were no significant differences between the patients and healthy subjects. Lines and ribbons represent the mean±sd. OTU: operational taxonomic unit.
