# Supplementary material for: Gut Microbial Dysbiosis Is Associated with Altered Hepatic Functions and Serum Metabolites in Chronic Hepatitis B Patients
Source: Front Microbiol. 2017 Nov 13;8:2222. doi: 10.3389/fmicb.2017.02222 (PMC5693892; doi:10.3389/fmicb.2017.02222)
Supplement: Supplementary file 3 [file Image3.PDF]

Supplementary Material

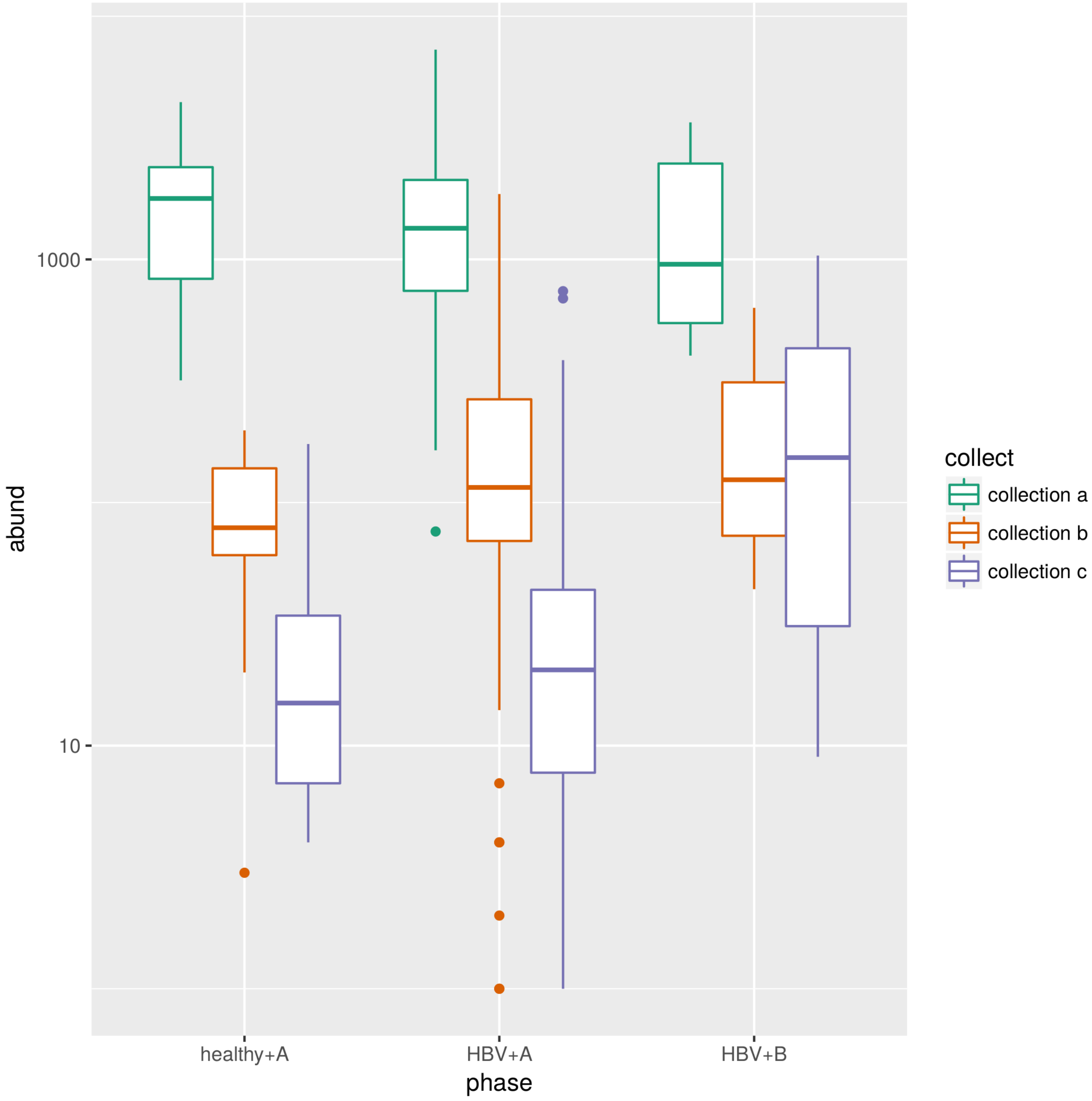

Supplementary Figure 3 Among the three collections of OTUs that were closely associated with serum metabolites, only collection c significantly was increased in CHB patients classified as phase B by Child-Pugh score. The abundance of each collection is the cumulative sum of the abundances of the OTUs belonging to that collection. OTU: operational taxonomic unit. CHB: chronic hepatitis B.
