# Supplementary material for: Gut Microbial Dysbiosis Is Associated with Altered Hepatic Functions and Serum Metabolites in Chronic Hepatitis B Patients
Source: Front Microbiol. 2017 Nov 13;8:2222. doi: 10.3389/fmicb.2017.02222 (PMC5693892; doi:10.3389/fmicb.2017.02222)
Supplement: Supplementary file 4 [file Image4.PDF]

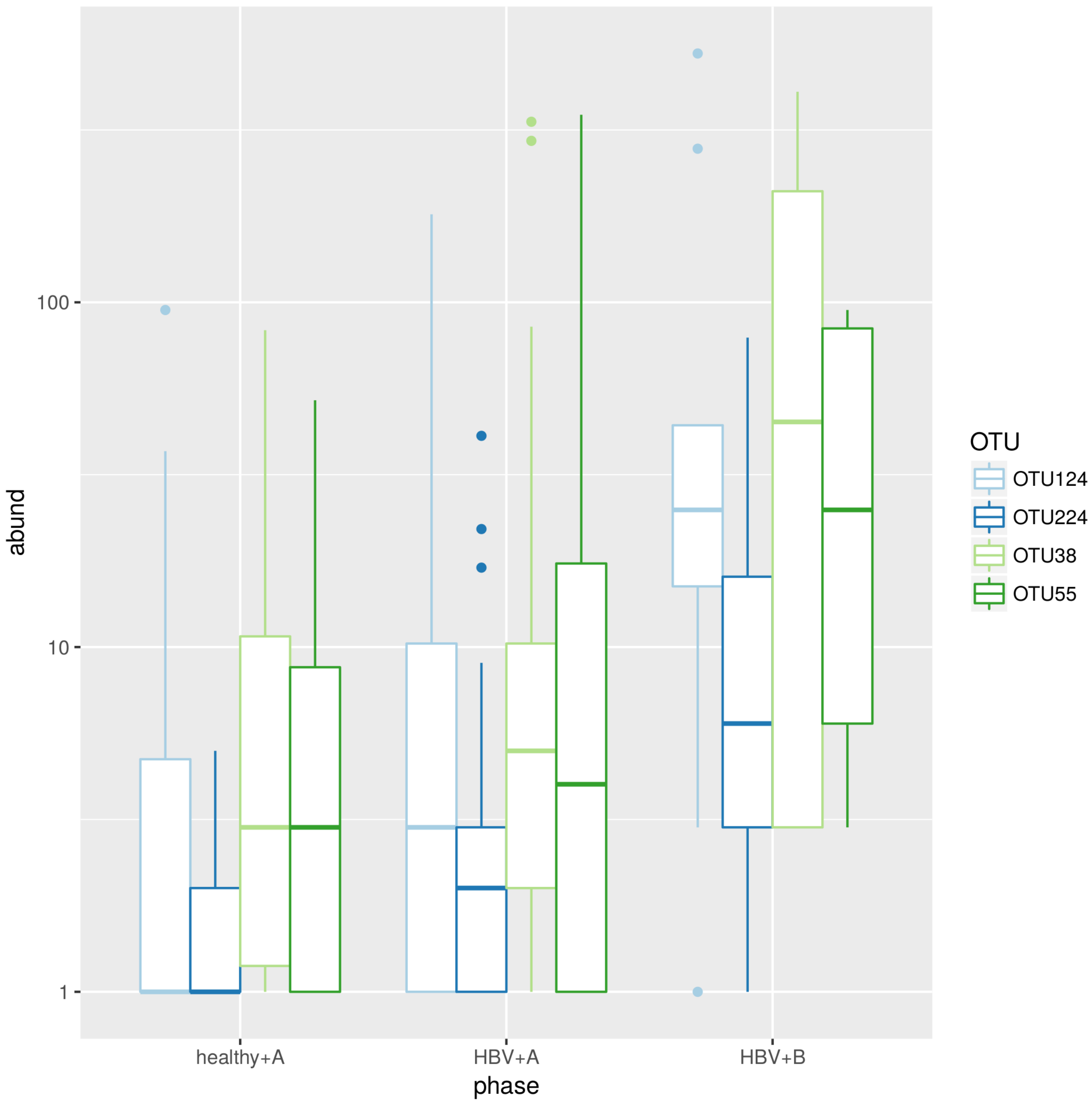

Supplementary Figure 4 The abundances of OTU55, OTU38, OTU224 and OTU124 were significantly increased in CHB patients classified as phase B by Child-Pugh score ($p<0.01$, Wilcoxon test). OTU: operational taxonomic unit. CHB: chronic hepatitis B.
