# Supplementary material for: Gut Microbial Dysbiosis Is Associated with Altered Hepatic Functions and Serum Metabolites in Chronic Hepatitis B Patients
Source: Front Microbiol. 2017 Nov 13;8:2222. doi: 10.3389/fmicb.2017.02222 (PMC5693892; doi:10.3389/fmicb.2017.02222)
Supplement: Supplementary file 5 [file Image5.PDF]

Supplementary Material

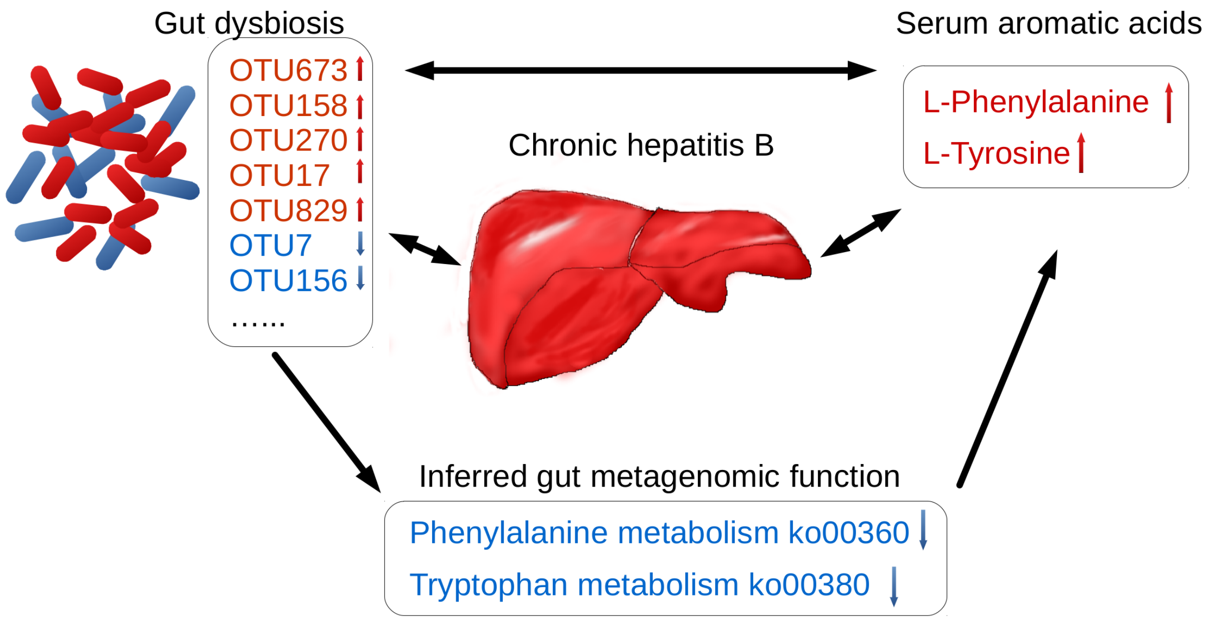

Supplementary Figure 5 The gut dysbiosis, the diminished AAA metabolisms in inferred metagenomic function and the accumulation of serum AAAs were closely interconnected and all associated with CHB. AAA: aromatic amino acid. CHB: chronic hepatitis B. OTU: operational taxonomic unit.
