# Supplementary material for: Gut Microbial Dysbiosis Is Associated with Altered Hepatic Functions and Serum Metabolites in Chronic Hepatitis B Patients
Source: Front Microbiol. 2017 Nov 13;8:2222. doi: 10.3389/fmicb.2017.02222 (PMC5693892; doi:10.3389/fmicb.2017.02222)
Supplement: Supplementary file 7 [file Table2.doc]

**Table S2. Temperature program of column incubator in serum sample**

| Rate  （℃/min） | Temperature  （℃） | Hold time  （min） |
| --- | --- | --- |
|  | 80 | 2 |
| 10 | 180 | 0 |
| 5 | 240 | 0 |
| 25 | 290 | 9 |
